# Smoking Cessation and Attempted Cessation among Adults in the United States

**DOI:** 10.1371/journal.pone.0093014

**Published:** 2014-03-27

**Authors:** Amir Goren, Kathy Annunziata, Robert A. Schnoll, Jose A. Suaya

**Affiliations:** 1 Health Outcomes Practice, Kantar Health, New York, New York, United States of America; 2 Health Outcomes Practice, Kantar Health, Princeton, New Jersey, United States of America; 3 Department of Psychiatry, University of Pennsylvania, Philadelphia, Pennsylvania, United States of America; 4 GlaxoSmithKline Vaccines, Philadelphia, Pennsylvania, United States of America; University of Granada, Spain

## Abstract

**Aims:**

With growing recognition of stagnant rates of attempted cigarette smoking cessation, the current study examined demographic and psychometric characteristics associated with successful and attempted smoking cessation in a nationally representative sample. This additional understanding may help target tobacco cessation treatments toward sub-groups of smokers in order to increase attempts to quit smoking.

**Design, setting, and participants:**

Data were used from the 2011 U.S. National Health and Wellness Survey (n = 50,000).

**Measurements:**

Current smoking status and demographics, health characteristics, comorbidities, and health behaviors.

**Findings:**

In 2011, 18%, 29%, and 52% of U.S. adults were current, former, or never smokers, respectively. Over one quarter (27%) of current smokers were attempting to quit. Current smokers (vs. others) were significantly more likely to be poorer, non-Hispanic White, less educated, ages 45–64, and uninsured, and they had fewer health-conscious behaviors (e.g., influenza vaccination, exercise). Attempting quitters vs. current smokers were significantly less likely to be non-Hispanic White and more likely to be younger, educated, insured, non-obese, with family history of chronic obstructive pulmonary disease, and they had more health-conscious behaviors.

**Conclusions:**

Smokers, attempting quitters, and successful quitters differ on characteristics that may be useful for targeting and personalizing interventions aiming to increase cessation attempts, likelihood, and sustainability.

## Introduction

Cigarette smoking is the leading cause of premature death, accounting for approximately 443,000 premature deaths [1], over $97 billion in lost productivity, and $96 billion in direct medical expenses [2] annually in the U.S. Current smokers have higher healthcare costs than never smokers [3], [4] and, compared with never smokers or former smokers, they experience greater work productivity loss and impairment [5]. In contrast, benefits of quitting smoking include quality of life and mortality rates (for those quitting prior to age 35) resembling those of never smokers [6], [7].

The rate of tobacco use in the U.S. has remained stable over the past decade [8]. The availability of only three types of medication for nicotine dependence and the poor utilization of these medications among U.S. smokers trying to quit are major reasons for the plateauing of cessation rates [9]. However, another critical reason for the stalling of progress in smoking cessation rates over the past decade is that the rate of attempted smoking cessation has also remained stagnant in the past decade [10]–[12]. Compared with previous years, smokers are generally showing a greater and greater reluctance to attempt to quit smoking. More importantly, very little is known about what characteristics define and distinguish those who attempt to quit smoking from those who have quit entirely or are not attempting to do so, in the general population.

Determining and distinguishing the characteristics of those who are attempting to quit or have quit successfully from those who are current smokers may help guide the implementation of tobacco cessation treatments in order to increase quit attempts. As most existing studies examine only current, former, and never smokers, the added focus on smokers who are attempting to quit in the current study can contribute meaningfully to our understanding of characteristics and obstacles associated with successful and attempted smoking cessation and may help generate interventions specifically for those who are attempting to quit. Boosting quit rates, in turn, may help reignite the steady decline in smoking rates seen prior to the past several years.

## Methods

### Study Population and Data Source

This study utilized the first two waves of the 2011 U.S. National Health and Wellness Survey (NHWS), an Internet-based, self-administered survey of healthcare attitudes and behaviors completed by a representative sample of U.S. adults (aged 18 or older), fielded during January to March and April to May of 2011. Data from all respondents were used to maximize power and representativeness of the results. The survey instrument was approved by the Essex Institutional Review Board (Lebanon, NJ). Survey participants were invited from Lightspeed Research (LSR), which maintains opt-in consumer panels and recruits panel members via email, e-newsletters, online banners, and co-registration with panel partners. LSR panel members are limited in the number of surveys they may complete during the year, and they receive points that can be accumulated and exchanged for prizes. Using a stratified random sampling framework, NHWS respondents were sampled within age, gender, and ethnicity strata so as to match the demographic composition of the U.S. adult population, as determined by the U.S. Census Bureau. The NHWS only allows missing data on a few sensitive questions; otherwise, respondents must answer all questions presented in order to complete the survey. Demographic composition and weighted prevalence estimates of various comorbid conditions in the NHWS have compared favorably with U.S. Census data and the National Health Interview Survey (NHIS) [13]–[15]. The NHWS data used in this study are made available for non-commercial research and validation purposes, upon request.

### Measures

#### Smoking group categories

In the NHWS, respondents who affirmed having ever smoked cigarettes were then asked, “Do you currently smoke cigarettes?” Respondents were classified in terms of their self-reported tobacco use on the basis of this question, as: (1) current smokers (“Yes, I smoke”), (2) attempting quitters (“Yes, but I am trying to quit”), and (3) former smokers (“No, I quit smoking” or, “No, I am in the process of quitting”).

#### Predictors

Self-reported demographic/health characteristics included: gender, age, race/ethnicity, marital status, employment, education, income, health insurance status, body mass index (BMI), exercise, taking steps to lose weight, use of the influenza vaccine, alcohol use, medical comorbidities (e.g., respiratory/cardiovascular disease), family history of chronic obstructive pulmonary disease (COPD), and the presence of children in the household. Due to lower rates of successful smoking cessation among certain populations (e.g., African Americans and those with mental illness), race/ethnicity measures were included [16]–[19].

### Statistical Analysis

#### Descriptive analyses

Analyses were used to describe characteristics of the total sample across smoking group categories. Prevalence of smoking group categories by selected demographic characteristics in the NHWS was compared with that in the NHIS to examine similarity of results between surveys. Missing categories (i.e., missing BMI based on declined reporting of height/weight and missing income) were included in all analyses in order to retain the full sample.

To better represent the demographic makeup of the U.S. adult population, results were weighted and projected by gender, age, race/ethnicity, and education in all bivariate and descriptive analyses, using Horvitz-Thompson sampling weights, which account for unequal probability of selection [20]. Post-stratification applied to NHWS sampling on the basis of the 2010 Current Population Survey (Annual Demographics File) of the U.S. Census Bureau helped to account further for over- or underrepresentation of specific segments of the population in the original sampling frame. Weighted prevalence estimates were provided for select subgroups of respondents, categorized by age (18–44, 45–64, and 65+) and race/ethnicity (Hispanic and non-Hispanic White and Black) groups.

#### Bivariate analyses

The aim was to identify variables for use in multivariate analysis and to identify weighted differences in respondent characteristics across smoking groups. Column proportion tests were used for categorical variables, and t-tests were used for continuous variables, with a two-tailed p-value <.05 used to indicate statistical significance.

#### Multivariable analysis

The aim was to determine respondent characteristics associated with smoking group categories. Multinomial logistic regression assessed demographic and health characteristics as predictors of the odds of membership in a group, relative to membership among current smokers. Certain odds ratios were inverted to assist interpretation.

Based upon results of the bivariate analysis or because certain variables (e.g., gender, age, race/ethnicity) are associated with smoking behavior [6], variables incorporated into multivariable analysis included: gender, age, race/ethnicity, marital status, employment, education, income, health insurance, BMI, exercise, taking any steps to lose weight, influenza vaccination, alcohol use, respiratory/cardiovascular comorbidity, mental health comorbidity, diabetes, headache/migraine, family history of COPD, and children in the household.

## Results

### Sample

The analyses included 50,000 respondents who consented to participate in and completed the NHWS. Excluded were those who refused to provide informed consent, were over the NHWS two-wave inclusion quota of 50,000 or specific age strata quotas, were under the minimum age of 18 years, or who otherwise quit during the survey. The overall completion response rate was 15.5% among qualifying respondents, or 73.2% completion among those who at minimum accessed the emailed invitation link to the survey informed consent page. Analyses included: 26,720 never smokers (i.e., those who reported never having smoked cigarettes: 53.4%); 5,923 current smokers (those who reported smoking currently: 11.8%); 2,215 attempting quitters (i.e., current smokers reporting that they were attempting to quit: 4.4%); and 15,142 former smokers (i.e., nonsmokers who quit: 30.3%).

### Descriptive Results

Projecting results to the U.S. adult population, over 42 and 66 million adults (or 18.5% and 29.1% of the U.S. adult population) were currently smoking or had quit smoking, respectively; and 52.4% of the population were never smokers (Table 1). Among all current smokers, 27.3% were attempting to quit (Table 2).

**Table 1:** Demographic characteristics of respondents age 18+ years by smoking status.

	2010 NHIS†			2011 NHWS		
		(n = 27,157)			(n = 50,000)	
	All current smokers‡	Former smokers‡‡	Never smokers‡‡‡	All current smokers§	Former smokers§§	Never smokers§§§
Unweighted sample size	5,147	5,737	16,083	8,138	15,142	26,720
Projected number of adults	44,113,885	49,472,964	134,405,737	42,184,197	66,493,664	119,681,015
Percentage of all adults	19%	22%	59%	18%	29%	52%
	Row percentage			Row percentage		
	[95% confidence interval]			[95% confidence interval]		
**Gender**						
Male (%)	21.5	25.5	53.0	20.5	30.8	48.7
	[20.6–22.4]	[24.5–26.5]	[51.8–54.2]	[19.9–21.1]	[30.1–31.5]	[48.0–49.5]
Female (%)	17.3	18.2	64.5	16.6	27.6	55.8
	[16.5–18.1]	[17.5–18.9]	[63.5–65.5]	[16.1–17.1]	[27.0–28.2]	[55.2–56.5]
						
**Age**						
18–44 years (%)	21.5	12.7	65.8	18.7	21.1	60.3
	[20.5–22.5]	[12.0–13.4]	[64.7–66.9]	[18.1–19.3]	[20.4–21.6]	[59.6–61.1]
45–64 years (%)	21.1	25.6	53.3	22.2	29.6	48.1
	[20.1–22.1]	[24.5–26.7]	[52.0–54.6]	[21.6–22.9]	[28.9–30.3]	[47.3–48.9]
65–74 years (%)	13.0	39.5	47.5	12.7	49.9	37.4
	[11.6–14.4]	[37.3–41.7]	[45.3–49.7]	[11.9–13.6]	[48.6–51.2]	[36.2–38.7]
75+ years (%)	5.1	39.2	55.7	6.9	52.9	40.2
	[4.0–6.2]	[36.9–41.5]	[53.4–58.1]	[5.7–8.4]	[50.3–55.4]	[37.7–42.8]
						
**Race/Ethnicity**						
Non-Hispanic White (%)	21.0	25.0	54.0	18.6	31.3	50.0
	[20.2–21.8]	[24.2–25.8]	[53.0–55.1]	[18.2–19.1]	[30.9–31.9]	[49.4–50.4]
Non-Hispanic Black (%)	20.6	14.0	65.5	19.4	21.5	59.1
	[19.2–22.0]	[12.8–15.2]	[63.8–67.2]	[18.1–20.7]	[20.1–23.0]	[57.5–60.8]
Hispanic or Latino (%)	12.5	14.7	72.9	18.1	26.1	55.8
	[11.4–13.6]	[13.5–15.9]	[71.4–74.4]	[16.7–19.6]	[24.5–27.8]	[53.9–57.6]
Non-Hispanic Asian (%)	9.2	12.6	78.2	12.1	18.5	69.4
				[10.8–13.6]	[16.6–20.5]	[67.2–71.5]
Non-Hispanic Other (%)	27.4	25.8	46.8	21.7	35.1	43.2
				[19.3–24.2]	[32.3–38.1]	[40.3–46.2]
**Region**						
Northeast (%)	17.4	23.3	59.3	18.3	29.3	52.3
	[16.0–18.8]	[21.7–24.9]	[57.4–61.2]	[17.4–19.3]	[28.3–30.4]	[51.2–53.5]
Midwest (%)	21.8	22.3	55.9	19.9	28.6	51.5
	[20.4–23.2]	[21.1–23.5]	[54.4–57.4]	[19.0–20.7]	[27.7–29.5]	[50.5–52.5]
South (%)	21.0	20.9	58.1	18.6	28.9	52.4
	[19.9–22.1]	[19.8–22.0]	[56.8–59.4]	[18.0–19.3]	[28.1–29.8]	[51.6–53.3]
West (%)	15.9	21.1	63.0	16.8	29.8	53.4
	[14.7–17.1]	[19.8–22.4]	[61.2–64.8]	[15.9–17.7]	[28.9–30.9]	[52.3–54.6]

Comparison between the 2010 National Health Interview Survey (NHIS) and the 2011 National Health and Wellness Survey (NHWS).

*Note*. Presented are row percentages (summing to 100% across columns), and in brackets are 95% confidence intervals for the row percentages. Data in this table are based on two questions in the NHIS: “Have you smoked at least 100 cigarettes in your entire life?” and “Do you now smoke cigarettes every day, some days, or not at all?”

† Source: Schiller et al., 2012 [15]. Race/ethnicity was recoded from variables (HISPAN_I; RACERPI2) to create mutually exclusive groups, summing to total adults. Percentages were from Table XV. Confidence intervals were manually calculated based on the standard errors as noted in Table XV; they could not be determined for non-Hispanic Asian and non-Hispanic Other, as standard errors were not available for these subgroups.

‡ Current smokers have smoked at least 100 cigarettes in their lifetime and still currently smoke. Every day smokers are current smokers who smoke every day, while some day smokers are current smokers who smoke on some days.

‡‡ Former smokers are persons who have smoked at least 100 cigarettes in their lifetime but currently do not smoke at all.

‡‡‡ Nonsmokers are persons who have never smoked at least 100 cigarettes in their lifetime.

§ Current smokers defined as those who responded, “Yes, I smoke” or, “Yes, but I am trying to quit.”

§§ Former smokers defined as those who responded, “No, I quit smoking” or, “No, I am in the process of quitting.”

§§§ Nonsmokers defined as those who responded, “No” to the question, “Have you ever smoked cigarettes?”

**Table 2:** Distribution of race/ethnicity and age group by smoking status based on the 2011 National Health and Wellness Survey (NHWS).

			Smoking group		
	Sample Size	Projected Adults	Current Smokers	Attempting Quitters	Former Smokers
Unweighted sample size	50,000	N/A	5,923	2,215	15,142
Adults age 18+ years	N/A	228,358,876	30,676,446	11,507,751	66,493,664
As a % of All	N/A	100%	13%	5%	29%
			Row percentage		
			[95% confidence interval]		
**All adults**					
Age 18–44 years (%)	21,528	110,054,959	13.3	5.3	21.0
			[12.8–13.9]	[5.0–5.7]	[20.4–21.6]
Age 45–64 years (%)	17,472	79,837,766	16.6	5.6	29.6
			[16.0–17.2]	[5.3–6.0]	[28.9–30.4]
Age 65+ years (%)	11,000	38,466,151	7.2	2.9	51.2
			[6.5–7.8]	[2.5–3.4]	[49.9–52.6]
Age 18+ years (%)	50,000	228,358,876	13.4	5.0	29.1
			[13.1–13.8]	[4.8–5.3]	[28.7–29.6]
**Hispanic**					
Age 18–44 years (%)	2,620	20,571,302	12.3	6.1	22.5
			[10.8–13.9]	[5.0–7.3]	[20.7–24.5]
Age 45–64 years (%)	1,119	8,400,293	15.2	5.3	28.9
			[12.9–17.8]	[4.0–7.1]	[25.9–32.1]
Age 65+ years (%)	245	2,674,120	6.2	2.4	44.6
			[3.3–11.3]	[1.0–5.5]	[37.0–52.6]
Age 18+ years (%)	3,984	31,645,715	12.6	5.6	26.1
			[11.4–13.9]	[4.8–6.5]	[24.5–27.8]
**Non-Hispanic White**					
Age 18–44 years (%)	13,293	66,958,092	14.4	5.2	22.4
			[13.7–15.0]	[4.8–5.7]	[21.7–23.2]
Age 45–64 years (%)	12,938	58,005,291	17.0	5.2	30.5
			[16.3–17.7]	[4.8–5.6]	[29.7–31.3]
Age 65+ years (%)	9,936	30,560,986	7.0	2.8	52.7
			[6.5–7.7]	[2.4–3.3]	[51.4–54.0]
Age 18+ years (%)	36,167	155,524,369	13.9	4.8	31.4
			[13.5–14.3]	[4.5–5.0]	[30.9–31.9]
**Non-Hispanic Black**					
Age 18–44 years (%)	2,726	14,191,753	11.9	5.0	13.3
			[10.5–13.5]	[4.0–6.1]	[11.9–14.9]
Age 45–64 years (%)	2,170	8,777,168	16.8	8.5	25.7
			[15.1–18.7]	[7.3–9.9]	[23.7–27.8]
Age 65+ years (%)	487	3,298,272	9.7	4.4	45.6
			[6.5–14.2]	[2.3–8.4]	[39.1–52.3]
Age 18+ years (%)	5,383	26,267,193	13.3	6.1	21.5
			[12.2–14.4]	[5.3–6.9]	[20.1–23.0]
**Non-Hispanic Other**					
Age 18–44 years (%)	2,889	8,333,812	10.2	5.1	18.8
			[8.9–11.7]	[4.2–6.2]	[17.2–20.5]
Age 45–64 years (%)	1,245	4,655,014	13.9	6.3	27.5
			[11.9–16.2]	[4.9–8.0]	[24.9–30.3]
Age 65+ years (%)	332	1,932,773	5.9	2.9	47.0
			[3.2–10.7]	[1.7–5.0]	[39.0–55.1]
Age 18+ years (%)	4,466	14,921,599	10.8	5.2	25.2
			[9.7–12.0]	[4.5–6.0]	[23.5–26.9]

*Note*. Non-Hispanic Other includes Asian and Other Races.

As seen in Table 1, proportions of current, former, and never smokers across gender, age, race/ethnicity, and geographic region categories identified in NHIS and NHWS were highly convergent [1]. The biggest disparity between NHWS and NHIS smoking prevalence was seen in the Hispanic figures: within NHWS, 18.1% of Hispanics were current smokers (including those attempting to quit) compared with 12.5% from the NHIS (Table 1).

In reviewing smoking status by age, NHWS showed the highest percentage of current smokers among ages 45–64 (16.6%). The highest proportion of former smokers was in the 65+ year age group (51.2%) (Table 2).

In terms of race/ethnicity, non-Hispanic Whites had the highest proportion of current (13.9%) and former (31.4%) smokers. The highest percentage of current smokers was among non-Hispanic Whites (17.0%) and Blacks (16.8%) aged 45–64, while the lowest was among Hispanics (6.2%) and non-Hispanic others (5.9%) aged 65+ (Table 2). The highest projected number of current smokers (almost 20 million adults) could be found among Whites ages 18–64.

### Bivariate Results

Current smokers, compared with attempting quitters and former smokers, were more likely to be male (54.5%), to have the lowest education levels (35.5% high school or less), lowest income levels, and least physical activity (Table 3).

**Table 3:** Comparison of characteristics across smoking groups based on the 2011 National Health and Wellness Survey (NHWS).

	Smoking group		
	Current Smokers	Attempting Quitters	Former Smokers
	(A)	(B)	(C)
Unweighted sample sizes	5,923	2,215	15,142
Adults age 18+ years	30,676,446	11,507,751	66,493,664
	Column percentage		
	[95% confidence interval]		
**Gender**			
Men	54.5^BC^	51.3	51.0
	[53.1–55.9]	[48.9–53.7]	[50.1–52.0]
Women	45.5^BC^	48.7	49.0
	[44.1–46.9]	[46.3–51.1]	[48.0–49.9]
**Age group**			
Age 18–44 years	47.8^BC^	51.1	34.8
	[46.4–49.3]	[48.7–53.5]	[33.9–35.7]
Age 45–64 years	43.2^BC^	39.1	35.6
	[41.8–44.6]	[36.9–41.4]	[34.7–36.5]
Age 65+ years	9.0^C^	9.8	29.6
	[8.2–9.8]	[8.5–11.3]	[28.8–30.6]
**Race/ethnicity**			
Hispanic	13.0^B^	15.3	12.4
	[11.8–14.2]	[13.2–17.6]	[11.6–13.3]
Non-Hispanic White/Caucasian	70.4^BC^	64.1	73.4
	[69.0–71.9]	[61.7–66.5]	[72.4–74.4]
Non-Hispanic African American	11.4^BC^	13.9	8.5
	[10.4–12.4]	[12.2–15.7]	[7.9–9.1]
Non-Hispanic Asian	2.3	3.2	2.5
	[2.0–2.7]	[2.6–3.9]	[2.2–2.8]
Non-Hispanic Others	2.9	3.6	3.2
	[2.5–3.4]	[2.9–4.4]	[2.9–3.5]
**Marital status**			
Married	40.1^C^	41.2	54.1
	[38.7–41.5]	[38.9–43.6]	[53.1–55.0]
Not married	59.9^C^	58.8	45.9
	[58.5–61.3]	[56.5–61.1]	[45.0–46.9]
**Employment status**			
Employed	55.7^C^	55.4	47.9
	[54.3–57.1]	[53.1–57.8]	[46.9–48.8]
Not employed	44.3^C^	44.6	52.1
	[42.9–45.7]	[42.2–46.9]	[51.2–53.1]
**Education**			
High School or less	35.5^BC^	29.8	25.0
	[34.1–36.9]	[27.6–32.1]	[24.2–25.9]
Some college, or Associate's degree	51.8^B^	54.7	51.2
	[50.4–53.2]	[52.3–57.0]	[50.2–52.1]
College degree or higher	12.7^BC^	15.5	23.8
	[12.0–13.5]	[14.3–16.7]	[23.1–24.5]
**Total annual household income**			
$0 to $24,999	29.2^BC^	26.6	18.8
	[27.9–30.5]	[24.4–28.8]	[18.1–19.7]
$25,000 to $49,999	32.9^C^	31.0	29.3
	[31.6–34.2]	[28.8–33.2]	[28.5–30.2]
$50,000 to $74,999	16.5^BC^	18.6	20.4
	[15.5–17.5]	[16.9–20.5]	[19.6–21.1]
≥$75,000	15.3^C^	16.8	22.2
	[14.3–16.3]	[15.2–18.6]	[21.5–23.0]
Decline to answer	6.2^C^	7.1	9.2
	[5.5–6.9]	[6.0–8.3]	[8.7–9.8]
**Have health insurance**	67.6^BC^	74.5	84.9
	[66.2–69.0]	[72.3–76.6]	[84.2–85.6]
**Body mass index (BMI)**			
Underweight (<19)	4.0^C^	3.4	1.7
	[3.5–4.6]	[2.7–4.3]	[1.5–2.0]
Normal (≥19 & <25)	33.5^C^	32.0	26.1
	[32.2–34.9]	[29.8–34.3]	[25.2–26.9]
Overweight (≥25 & <30)	31.5^C^	31.6	33.2
	[30.2–32.9]	[29.5–33.8]	[32.3–34.1]
Obese (≥30)	30.9^C^	33.0	39.0
	[29.6–32.3]	[30.8–35.3]	[38.1–40.0]
**Mean days exercised in the past month**	5.6^BC^ (SD = 8.25)	7.3 (SD = 8.71)	7.6 (SD = 8.91)
**Weight control**			
Taken steps to lose weight	34.7 ^BC^	53.6	55.4
	[33.3–36.0]	[51.1–55.9]	[54.5–56.4]
**Flu vaccine in the past year**	26.9^BC^	34.8	44.3
	[25.7–28.2]	[32.6–37.0]	[43.4–45.3]
**Drink alcohol**	72.5^C^	74.1	68.7
	[71.2–73.7]	[72.0–76.1]	[67.8–69.6]
**Comorbidities**			
Respiratory/cardiovascular†	32.3^BC^	37.4	43.5
	[31.0–33.7]	[35.1–39.7]	[42.5–44.4]
Mental health‡	43.5^BC^	49.2	33.3
	[42.0–44.9]	[46.8–51.6]	[32.5–34.3]
Diabetes	10.1^C^	9.3	14.6
	[9.3–11.0]	[8.0–10.7]	[13.9–15.3]
Headache/migraine‡‡	17.5^BC^	21.4	15.3
	[16.5–18.6]	[19.5–23.4]	[14.6–16.0]
**Family history of COPD**	5.9^BC^	7.9	4.3
	[5.3–6.6]	[6.8–9.2]	[4.0–4.7]
**Living with children under 18 years of age**	35.0^C^	36.9	26.1
	[33.6–36.4]	[34.5–39.2]	[25.2–26.9]

*Note*. Superscripts represent columns whose percentage is significantly different from the column (A) at p<.05. The reference group for statistical tests was column A, Current Smokers. Superscripts for significant differences across columns B–C only are not shown. SD  =  standard deviation.

† Respiratory/cardiovascular conditions include diagnoses of any of the following: COPD, asthma, emphysema, chronic bronchitis, hypertension, and heart attack.

‡ Mental health conditions include any of the following: diagnoses of bipolar disorder, depression, GAD, anxiety, and OCD; and experiences of insomnia and alcoholism.

‡‡ Headache/migraine conditions include diagnoses of any of the following: headache and migraine.

Former smokers were the oldest segment (29.6% at age 65+) and the most likely to be retired or otherwise not employed (52.1%); they were the most likely to be married (54.1%); and 39.0% of former smokers were obese (vs. less than a third of current smokers or those attempting to quit). They had the highest rate of respiratory/cardiovascular comorbidities (43.5%), were the most likely to have received a flu vaccination in the past year (44.3%), and were least likely to have children in the household.

Most respondents attempting to quit were non-Hispanic whites (64.1%),a lower proportion than among current or former smokers. Relative to current smokers, those attempting to quit were more likely to be women (48.7% vs. 45.5%) and to have a college degree or higher (15.5% vs. 12.7%). Those attempting to quit exercised more often than current smokers (7.3 days vs. 5.6 days), and were more likely to have been vaccinated for the flu in the past year (34.8% vs. 26.9%). Those attempting to quit were the most likely to have mental health comorbidities (49.2%) and headaches/migraines (21.4%). Current smokers and those attempting to quit were the most likely to drink alcohol.

### Multivariate Results

Multinomial logistic regression results are broken out by never smokers, attempting quitters, and former smokers for ease of interpretation and comparison to the literature. All results (Table 4) are presented with current smokers as the reference group. “Decline to answer” responses were analyzed (in order to retain the full sample) but are not interpreted further.

**Table 4:** Adjusted odds ratios of smoking group membership as a function of demographics, comorbidities, and health characteristics and behaviors.

	Smoking group membership		
	Attempting quitters	Former Smokers†	Never smokers
	(n = 2,215)	(n = 15,142)	(n = 26,720)
**Gender**			
Male (vs. female)	1.03	1.00	**0.73** *
	[0.93–1.15]	[0.93–1.07]	[0.68–0.78]
**Age group**			
Age 45–64 years (vs. 18–44 years)	**0.80** *	**0.87** *	**0.64** *
	[0.71–0.91]	[0.80–0.94]	[0.59–0.69]
Age 65+ years (vs. 18–44 years)	0.93	**2.38** *	0.92
	[0.77–1.12]	[2.12–2.68]	[0.82–1.03]
**Race/ethnicity**			
Hispanic (vs. non-Hispanic White)	**1.32** *	**1.21** *	**1.32** *
	[1.10–1.59]	[1.07–1.38]	[1.18–1.49]
Non-Hispanic Black (vs. non-Hispanic White)	**1.44** *	**0.86** *	**1.30** *
	[1.22–1.68]	[0.77–0.96]	[1.18–1.44]
Other race/ethnicity (vs. non-Hispanic White)	**1.45** *	1.13	**1.34** *
	[1.20–1.74]	[1.00–1.29]	[1.19–1.50]
**Marital status**			
Married/partnered (vs. single)	0.99	1.07	**0.77** *
	[0.86–1.14]	[0.98–1.17]	[0.70–0.83]
Divorced/separated/widowed (vs. single)	1.01	**0.80** *	**0.48** *
	[0.86–1.18]	[0.72–0.88]	[0.43–0.53]
**Employment status**			
Employed (full-/part-time or self-employed vs. unemployed)	0.93	**0.90** *	1.05
	[0.84–1.04]	[0.84–0.97]	[0.98–1.12]
**Education**			
High school or less (vs. greater)	**0.82** *	**0.65** *	**0.51** *
	[0.73–0.92]	[0.61–0.71]	[0.48–0.55]
**Total annual household income**			
Income: $25K to <$75K (vs. <$25K)	1.06	**1.29** *	**1.27** *
	[0.93–1.21]	[1.19–1.41]	[1.18–1.38]
Income: $75K+ (vs. < $25K)	1.10	**1.72** *	**2.21** *
	[0.92–1.31]	[1.54–1.92]	[1.99–2.45]
Income: Decline to answer (vs. <$25K)	**1.33** *	**1.76** *	**2.13** *
	[1.07–1.65]	[1.53–2.03]	[1.86–2.42]
**Health insurance**			
Insured (vs. uninsured)	**1.23** *	**1.59** *	**1.64** *
	[1.09–1.40]	[1.46–1.73]	[1.52–1.76]
**Body mass index (BMI)**			
BMI: Overweight (vs. normal-underweight)	0.93	**1.15** *	1.02
	[0.82–1.06]	[1.06–1.25]	[0.94–1.10]
BMI: Obese (vs. normal-underweight)	**0.87** *	**1.48** *	**1.19** *
	[0.76–1.00]	[1.36–1.62]	[1.10–1.29]
BMI: Decline to answer (vs. normal-underweight)	0.71	**1.49** *	**1.61** *
	[0.41–1.24]	[1.08–2.06]	[1.20–2.17]
**Regular physical activity**			
Exercise (20+ min, 12+ times/month, vs. less)	**1.39** *	**1.54** *	**1.63** *
	[1.24–1.56]	[1.43–1.66]	[1.51–1.75]
**Weight control**			
Taken steps to lose weight (vs. none)	**2.08** *	**2.03** *	**1.70** *
	[1.86–2.32]	[1.89–2.18]	[1.59–1.82]
**Flu vaccine in past year**			
Yes (vs. none)	**1.28** *	**1.51** *	**1.40** *
	[1.15–1.43]	[1.41–1.63]	[1.31–1.50]
**Alcohol**			
Drink (≥2–3 times per week, vs. <2 times)	0.92	**0.75** *	**0.40** *
	[0.82–1.03]	[0.69–0.80]	[0.37–0.43]
**Comorbidities**			
Respiratory/cardiovascular (vs. none)§	**1.13** *	**1.13** *	**0.88** *
	[1.01–1.27]	[1.05–1.21]	[0.82–0.94]
Mental health (vs. none)§§	**1.21** *	**0.71** *	**0.46** *
	[1.09–1.34]	[0.66–0.76]	[0.43–0.49]
Diabetes (vs. none)	**0.79** *	0.93	**0.76** *
	[0.67–0.94]	[0.84–1.03]	[0.69–0.84]
Headache/migraine (vs. none)§§§	1.10	0.97	**0.86** *
	[0.96–1.25]	[0.89–1.06]	[0.79–0.94]
**Family history of COPD**			
Yes (vs. no)	**1.31** *	**0.71** *	**0.50** *
	[1.07–1.59]	[0.61–0.82]	[0.44–0.58]
**Living with children under 18 years of age**			
Yes (vs. none)	1.04	**0.80** *	**0.92** *
	[0.92–1.17]	[0.74–0.87]	[0.86–0.99]

*Note*. Presented are odds ratios (and in brackets 95% confidence intervals for the odds ratios) obtained from a multinomial logistic regression, representing the relative odds of being in the group indicated vs. being in the reference group of current smokers (n = 5,923). The multinomial logistic regression had a significant overall fit, with χ2 = 10361 (df = 81), *p*<.001, and with a −2 log likelihood of 81154.

* *p*<.05.

† Former smokers include those who have quit smoking, as well as those who are not currently smoking because they are in the process of quitting.

§ Respiratory/cardiovascular conditions include diagnoses of any of the following: COPD, asthma, emphysema, chronic bronchitis, hypertension, and heart attack.

§§ Mental health conditions include any of the following: diagnoses of bipolar disorder, depression, GAD, anxiety, and OCD; and experiences of insomnia and alcoholism.

§§§ Headache/migraine conditions include diagnoses of any of the following: headache and migraine.

#### Never smokers

Relative to never smokers, current smokers were more likely to be: male; 45–64 years old; non-Hispanic White; non-single; educated beyond high school; poorer; uninsured; normal or underweight vs. obese; not exercising regularly; not taking steps to lose weight; not vaccinated for influenza; using alcohol at least twice per week; at greater risk of comorbidity; from a family with a history of COPD; and living with children in the household.

#### Attempting quitters

Compared with current smokers, those who were attempting to quit were more likely to be: 18–44 years old; any race/ethnicity other than non-Hispanic White; educated beyond high school; insured; normal-underweight vs. obese; exercising regularly; taking steps to lose weight; vaccinated for influenza; diagnosed with a respiratory or mental health comorbidity but not as likely diagnosed with diabetes; and with a family history of COPD.

#### Former smokers

Compared with current smokers, those who quit were more likely to be: 18–44 years old (vs. 45–64) or 65+ (vs. 18–44); Hispanic (but less likely non-Hispanic Black) vs. non-Hispanic White; single vs. divorced/separated/widowed; unemployed; educated beyond high school; wealthier; insured; overweight or obese vs. normal-underweight; exercising regularly; taking steps to lose weight; vaccinated for influenza; not using alcohol; at higher risk of respiratory but lower risk of mental health comorbidity; without a family history of COPD; and not living with children.

## Discussion

The present study with a nationally representative sample was unique in its inclusion of individuals who were attempting to quit, in addition to those who continued to smoke, had never smoked, or had successfully quit, as well as its inclusion of a broad range of predictors. This extends one other similar investigation using a national sample [21], which also examined predictors of attempted cessation; however, the current study investigates a new sample and utilizes an expanded set of potential predictors that included numerous comorbid conditions and, notably, health behaviors. This sample and data analytic approach were used to better understand the ways in which smokers may be helped to increase their willingness to make a quit attempt, providing an important way to reignite the steady decline in smoking rates seen prior to the past decade. Several notable findings emerged from our analyses.

First, the NHWS study results, in terms of proportions of Americans classified as current, former, or never smokers, align closely with many of the results from the National Health Interview Survey (NHIS) [15]. This supports the relevance and validity of the NHWS data source in profiling U.S. adults by their current smoking status.

Second, the multivariate models revealed a range of potential differences between our groups of current smokers, attempting quitters, and former smokers. Current smokers (vs. other groups) tended to be poorer, non-Hispanic White, less educated, more likely of age 45–64, uninsured, and with fewer health-conscious behaviors (e.g., influenza vaccination, exercise) and attempting quitters (vs. current smokers) were younger, less likely non-Hispanic White, more educated, insured, less frequently obese, more frequently taking steps to be health conscious, and with family history of COPD. Indeed, those with a high school education or less had the highest adjusted odds of being current smokers. This finding is consistent with prior research [1] and underscores that lower educational attainment is associated not only with a greater likelihood of smoking but also a lower likelihood of quit attempts [22], [23].

Whereas non-Hispanic Blacks were at greater odds of attempting to quit (along with Hispanics) than non-Hispanic Whites, they had lower odds of being quitters. These results are consistent with other research showing that, while African Americans are more likely to try to quit than non-Hispanic Whites, they are less likely to achieve long-term abstinence [18], [24]. This result highlights the need to develop novel intervention strategies to capitalize on the relatively higher motivation to quit among African Americans.

Women were more likely than men to have never smoked, but gender was not associated with the likelihood of attempting to quit smoking. Past studies, involving data from randomized clinical trials, have indicated that women are more likely than men to try to quit but are less likely to do so successfully [19], [23]. But the present national survey data suggest that attempts to quit smoking are equal across men and women. Since clinical trial data are restricted to the inclusion of treatment-seeking smokers, perhaps the present data are more representative of the general population of smokers.

Both older and younger respondents are represented among those who quit smoking (vs. current smokers). These results are consistent with research showing that older respondents are more likely to quit successfully [25]. Importantly, age was adjusted for in multivariate analysis, ensuring that this characteristic is not confounding the remaining results. However, aside from the older ex-smokers, younger respondents (18–44 year-olds) were more likely to be attempting quitters or former smokers than current smokers. This represents a hopeful sign that the younger generations are more open to quitting than perhaps previously shown [18], [22].

A family history of COPD was also associated with a greater likelihood of attempting to quit smoking, which has been suggested by past research [26], [27]. This result could be used to reconcile the findings of increased comorbidity, especially respiratory disease, among attempting and former smokers in the current study with past studies showing decreased comorbidity with cessation [28]–[31]. The seemingly paradoxical findings highlight how the current study is better suited to examining how respondent characteristics differ naturally across smoking groups, in contrast to longitudinal studies. Quitters and attempting quitters in the current sample appear to have had strong motivating reasons to quit, including greater respiratory and cardiovascular comorbidities, which may have been reduced over time, but not to the levels of current or never smokers who never experienced such comorbidity. Some of the increased comorbidity in the current study may also reflect physical health status declines associated with aging (adjustments for age notwithstanding). Future comparisons should stratify by age, especially if comparing long-term quitters against older current smokers.

Former smokers (vs. current smokers) were more health conscious, mentally healthy, and educated, but they were also at greater odds of obesity than current smokers and were more likely taking steps to lose weight. These findings are consistent with prior findings showing an average weight gain of over 9 pounds within 10 years of smoking cessation [29], [32] and may reflect a trajectory of weight gain over many years since quitting. It is also worth noting that never smokers were more likely to be obese than current smokers, consistent with the use of tobacco to manage weight.

With respect to comorbidities, those who had mental health comorbidities or a family history of COPD were also more likely to be attempting to quit smoking than current smokers, but at the same time, they were generally less likely to be quitters than current smokers. The mental health and alcohol use findings are consistent with previous research suggesting that mental health problems and alcohol abuse are associated with greater likelihood of smoking and lower quit rates [33], [34]. However, mental health conditions such as depression may be associated with an unfulfilled attempt to quit—a possibility that needs to be examined in longitudinal research but is an intriguing supplement to existing research focused on sustained quitters (i.e., that cessation is associated with a reduction in psychiatric comorbidity) [35], [36]. Lastly, the results concerning diabetes (associated with lower odds of attempting to quit) suggest that smoking cessation efforts need to be prioritized toward this sub-group.

Respondents who received flu vaccinations, exercised, took steps to lose weight, and used alcohol infrequently, were less likely to be current smokers. These findings contribute to the literature on health attitudes that characterize those who are unlikely to become or remain smokers [12], [37], as well as health behaviors (e.g., lower alcohol intake) and environments (smoke-free households) that can play a similar role in promoting abstinence [18], [25].

Finally, in considering an approach to intervening with smokers to promote quit attempts, lack of insurance coverage may be a notable barrier. In the present analysis, current smokers were less likely to be insured than attempting quitters or quitters, suggesting that removing barriers to healthcare access may help motivate quit attempts and success. This finding converges with evidence indicating that insurance coverage for smoking cessation interventions can help increase quit rates [38].

The current study examined a relatively heterogeneous sample of current smokers and attempting quitters. For example, 7.2% [CI: 6.1–8.5%] of attempting quitters reported currently using prescription medication to aid in their smoking cessation, and 11.2% [CI: 9.9–12.7%] used an over-the-counter cessation medication. Thus, the majority (over 80%) of attempting quitters were not using any pharmacological therapy in their cessation efforts. Additionally, levels of nicotine dependence varied, as shown via Fagerström Test for Nicotine Dependence (FTND) scores [39]. Among attempting quitters, 53.7% [CI: 51.3–56.1%] exhibited low to moderate dependence (scores of 1–4), and 26.8% [CI: 24.8–29.0%] exhibited high dependence (scores of 5 or higher). However, 19.5% [CI: 17.6–21.5%] had a score of 0, indicating no meaningful dependence. Mean dependence overall for attempting quitters was 3.0 (SD = 2.31). By comparison, among current smokers, 11.5% [CI: 10.6–12.5%] exhibited no meaningful dependence, 46.0% [CI: 44.5–47.4%] exhibited low to moderate dependence, and 42.6% [CI: 41.2–44.0%] exhibited high dependence, with mean dependence at 4.0 (SD = 2.45). The higher rates and level of dependence among smokers vs. attempting quitters is in line with expectations that nicotine dependence is a barrier to smoking cessation. Other sources of heterogeneity (e.g., duration of smoking, number of quit attempts) were not assessed in the NHWS, and the specific patterns of variance among these factors could contribute to the pattern of characteristics seen to differ across groups in this particular subsample of the population.

Causal conclusions cannot be drawn from this cross-sectional survey. Furthermore, there may be biases associated with self-report data, although there is some evidence for reliability and validity of self-report smoking data [40]. Additionally, the NHWS does not include adults who are unable to complete an online survey. The completion response rate (15.5%) suggests that additional selection biases may be present, although this response rate is similar to or higher than response rates of 15% [41] and 4.5% [42] observed in other online smoking cessation studies. However, it is difficult to assess the precise nature of the bias or the degree to which this response rate differs from other (online) surveys, as several contributing factors differ across types of surveys, recruitment methods, and target populations. For example, the majority of NHWS non-respondents did not access the link in the invitation email (which contained minimal, if any, information about the nature of the survey), and therefore, excluded respondents may have opted out only for very general reasons such as lack of time and interest in participating in any survey at the time (a limitation that applies to online panels generally). More clearly relevant to self-selection concerns is the considerably higher, 73.2% completion rate among those who accessed the survey and therefore had an opportunity to read about the nature of the NHWS and its expected 45-minute completion time. Those who were neither motivated nor able to participate in a survey of such length and/or topic—note that the NHWS draws respondents from a general, not healthcare-specific, panel population—would have refused or quit during informed consent or otherwise quit during the main survey. These factors and others (e.g., adult target population, non-academic setting), regardless of comparability to other online studies, may limit the generalizability of the current findings to those who have relatively better access to technology, are not institutionalized or otherwise impaired to the extent that they cannot participate, and are otherwise motivated and have the time to complete a comprehensive health survey. Furthermore, along with the age differences seen across groups, it is important to note the probability of cohort differences: former smokers may represent many older smokers from a different cohort than current smokers or attempting quitters, and they may have been exposed to cultural and media influences that differed across generations with respect to the harm of smoking and the need for cessation.

Nevertheless, this study has several novel findings and suggests areas warranting further investigation. Potential quitters resemble successful quitters in some ways (e.g., more health-conscious attitudes and behaviors, better education) but exhibit unique characteristics that may motivate their immediate attempts or hinder successful cessation. Mental health comorbidities may hinder their efforts to make a quit attempt and being non-Hispanic Black may yield systemic barriers (e.g., lower access to health insurance) that prevent successful cessation in spite of a willingness to quit. This suggests the need for multifaceted smoking cessation interventions that target smoking, comorbidities, and other barriers (e.g., lack of coverage) that smokers may be experiencing, in order to yield higher quit attempts and an increased likelihood that such attempts will be successful.

A second contribution of the study is its findings regarding age and respiratory/cardiovascular comorbidities. Interpreted in combination with extant research, the findings suggest that younger smokers under the age of 45 are much more likely to attempt to quit and to do so successfully, but if they are relatively healthy, they may not remain abstinent beyond the first few years. Respiratory/cardiovascular comorbidities may help drive long-term abstinence among both younger and older former smokers. Future research can help identify and define subgroups such as these more clearly.

In summary, the range of characteristics identified in this study, especially among attempting quitters, can help healthcare providers target the most effective smoking interventions at those who are most likely to benefit from them. Such targeted interventions are rendered more feasible with an enhanced understanding of the interaction between smoker characteristics, treatment characteristics, and health drivers of smoking cessation. Targeted interventions can reignite past reductions in U.S. smoking rates.
